# Supplementary material for: A framework for evaluating predicted sperm trajectories in crowded microscopy videos
Source: PLoS Comput Biol. 2026 Feb 10;22(2):e1013955. doi: 10.1371/journal.pcbi.1013955 (PMC12912684; doi:10.1371/journal.pcbi.1013955)
Supplement: S1 Appendix — (PDF) [file pcbi.1013955.s001.pdf]

S1 Appendix. Mathematical Formulation of Common Cell Tracking Metrics

For convenience of the reader, we provide a description of the common multi-object and cell tracking metrics. Relevant works are cited to provided sources for additional details.

Association Accuracy and Target Effectiveness

Association Accuracy (AA) and Target Effectiveness (TE) have been a standard metric within the cell tracking literature. AA is a measure of the number of correctly predicted cell associations between frames divided by the total number of associations in the ground truth data. TE is a measure describing the number of frames a ground truth track is correctly followed by the predicted tracks. These two metrics are still used frequently to compare modern cell tracking approaches [1, 2, 3].

MOTA, IDF1, HOTA, and CHOTA

Multi-object tracking has many applications outside the realm of cell tracking. Standard metrics have been used for comparing methods on various tasks, such as in the Multi-object Tracking Benchmark [4]. These metrics have also been adopted as cell tracking for comparison, such as in the Cell Tracking With Mitosis Detection Dataset Challenge (CMTC) [5].

MOTA is a measure of the total detection coverage, and is calculated as

$$MOTA = 1 - \frac{\sum_t FN_t + FP_t + IDSW_t}{\sum_t GT_t} \tag{1}$$

where t is given frame of the video, GT is the number of objects in the ground truth of the frame, FN is the number of false negatives (missed detections), FP is the number of false positives (spurious detections), and $IDSW$ is number of correct detections that are labeled with a different ID than the track they were associated with in the previous frame. The best MOTA score is 1.0 and scores can go into the negatives.

IDF1 is a measure of the consistency of labels across frames of a video. It uses the Hungarian algorithm to optimize how long each ground truth track was connected to a single ID trajectory. After this matching, the metric is calculated as

$$IDF1 = \frac{2IDTP}{2IDTP + IDFP + IDFN} \tag{2}$$

where IDTP is the total length and number of correctly labeled tracks for all frames, IDFP is the number of predicted tracks that don't match any ground truth, and IDFN is ground truth tracks that weren't predicted.

Recently, a new metric has been proposed called HOTA that better balances detection and association pieces [6]. A variant of this metric has been proposed specifically for cell tracking called CHOTA [7], but CHOTA was designed specifically to measure mitosis splitting events which do not occur in sperm.

DET, LNK, TRA, and TF

The Cell Tracking Challenge [8] has adopted standard evaluation methodology that can be used across many types of cells and data types. Many of these metrics rely on the Acyclic Oriented Graph Matching (AOGM) measure that is presented in [9]. This measure determines the number of steps needed to correct a graph representation of the predicted tracks to match the graph representation of the ground truth tracks. It is calculated as

$$AOGM = w_{NS}NS + w_{FN}FN + w_{FP}FP + w_{ED}ED + w_{EA}EA + w_{EC} \tag{3}$$

In this formulation, FN and FP represent false negatives and false positive in detections. ED, EA, and EC represent different kinds of incorrect tracking associations (edges in the graph). w represents a weight applied to each measure based on how difficult it would be to correct an error through a manual modification. The standard values are $w_{NS} = 5, w_{FN} = 10, w_{FP} = 1, w_{ED} = 1, w_{EA} = 1.5, w_{EC} = 1$. NS represents mitosis events where cell splits, so this term can be ignored for sperm tracking.

The DET, LNK, and TRA metrics derive from this measure. As an example, TRA is calculated as

$$TRA = 1 - min(AOGM, AOGM_0)/AOGM_0 \tag{4}$$

where $AOGM$ is the value of the metric for the predicted tracks, and $AOGM_0$ is the value of the metric for an empty reference (i.e. creating all tracks from scratch). This forces the measure to be between 0-1. DET is calculated in the same way but with no weight assigned to the edge calculations ($w_{ED} = w_{EA} = w_{EA} = 0$). Likewise, LNK is calculated with no weight assigned to the node calculations ($w_{NS} = w_{FN} = w_{FP} = 0$).

The Cell Tracking Challenge [8] also presents other biological plausibility metrics, many related to cell mitosis. Track Fractions (TF) is of particular interest for the sperm tracking problem. For each ground truth track, the predicted track that matches for the most frames is determined. Then, the number of frames of that are correctly matched is divided by the total frames for the ground truth track. These are then averaged for all ground truth sperm trajectories.

Jaccard Similarity/IoU/SEG

Whether as a bounding box or a pixel segmentation, the accuracy of the overlap between predicted and ground-truth regions is usually calculated using Jaccard Similarity. This is also known as Intersection over Union (IoU). This is calculated as

$$IoU = \frac{R \cap S}{R \cup S} \tag{5}$$

where R is the ground truth segmentation and S is the predicted segmentation. The SEG measure from the Cell Tracking Challenge [8] is also based-on this score. In this work, we do not provide segmentation results do due the difficulty of manually labeling each pixel. Thus, this metric is not presented in this work.

References

[1] Hayashida J, Nishimura T. MPM: Joint Representation of Motion and Position Map for Cell Tracking. Proceedings of the IEEE/CVF Conference on Computer Vision and Pattern Recognition. 2020;.

[2] Nishimura K, Hayashida J, Wang C, Ker DFE, Bise R. Weakly-Supervised Cell Tracking via Backward-and-Forward Propagation. In: Computer Vision – ECCV 2020: 16th European Conference, Glasgow, UK, August 23–28, 2020, Proceedings, Part XII. Berlin, Heidelberg: Springer-Verlag; 2020. p. 104–121. Available from: https://doi.org/10.1007/978-3-030-58610-2_7.

[3] Ben-Haim T, Riklin-Raviv T. Graph Neural Network for Cell Tracking in Microscopy Videos. In: Proceedings of the European Conference on Computer Vision (ECCV); 2022.

[4] Dendorfer P, Osep A, Milan A, Schindler K, Cremers D, Reid I, et al. MOTChallenge: A Benchmark for Single-camera Multiple Target Tracking. International Journal of Computer Vision. 2020; p. 1–37.

[5] Anjum S, Gurari D. CTMC: Cell Tracking With Mitosis Detection Dataset Challenge. In: Proceedings of the IEEE/CVF Conference on Computer Vision and Pattern Recognition (CVPR) Workshops; 2020.

[6] Luiten J, Osep A, Dendorfer P, Torr P, Geiger A, Leal-Taixé L, et al. Hota: A higher order metric for evaluating multi-object tracking. International journal of computer vision. 2021;129:548–578.

[7] Kaiser T, Ulman V, Rosenhahn B. CHOTA: A Higher Order Accuracy Metric for Cell Tracking. In: European Conference on Computer Vision Workshops (ECCVW). Springer; 2024.

[8] Maška M, Ulman V, Delgado-Rodriguez P, Gómez de Mariscal E, Necasova T, Guerrero Peña FA, et al. The Cell Tracking Challenge: 10 years of objective benchmarking. Nature Methods. 2023;20:1–11. doi:10.1038/s41592-023-01879-y.

[9] Matula P, Maška M, Sorokin DV, Matula P, Ortiz-de Solórzano C, Kozubek M. Cell tracking accuracy measurement based on comparison of acyclic oriented graphs. PloS one. 2015;10(12):e0144959.
