# Supplementary material for: A framework for evaluating predicted sperm trajectories in crowded microscopy videos
Source: PLoS Comput Biol. 2026 Feb 10;22(2):e1013955. doi: 10.1371/journal.pcbi.1013955 (PMC12912684; doi:10.1371/journal.pcbi.1013955)
Supplement: Attachment — Submitted filename: ResponseToReviewers.docx [file pcbi.1013955.s003.docx]

We thank the reviewers for their time and their feedback. We wish to address each point of feedback and show how it is incorporated into our revised manuscript.

# Reviewer 1 Feedback

Reviewer 1’s original comments are provided in italics for convenience:

- *The topic of your research is interesting and original. There are, however, some comments* *mainly about the organization of the manuscript. The experimental design of the study is not described in the materials and methods part, but it seems that this information is included in the results part.*

Reviewer 1 then pointed out many organizational improvements that could be made to the document, specifically with pulling more material into the methodology section. We have analyzed the document to see which methodology components are present in the results section that can be moved. Specifically, we address the following sections that were brought up.

- *Baseline Tracker and Dataset (L132)*

Information on the baseline tracker was moved from the Results section to the Methods section with heading **Baseline Tracker and Labeling.** Dataset information is now included as part of the **Data Collection** heading in the Methods section.

- *Experiment 1: A Poorly Configured Tracker (L152),*
- *Experiment 2: Short vs. Long Timescale Trajectories (L177),*
- *Experiment 3: Bounding Boxes vs. Centroids (L204),*
- *“We also output Earth Mover’s Distance (EMD) scores…”*

We extracted various information related to these sections and placed it in the methodology. Specifically, it focuses on how the kinematic parameters are calculated, visualized, and compared for the three experiments. This is now present in a subsection with heading **Analysis of Tracking Algorithms - Kinematic Parameter Distributions.** For convenience, it is copied in full below.

……………………..

**Analysis of Tracking Algorithms - Kinematic Parameter Distributions**

Semen characteristics are typically analyzed using a small set of standard kinematic parameters. These outputs come from Computer-Aided Semen Analysis (CASA), typically performed using commercial hardware/software systems. CASA is often used to define and classify sperm motility and movement patterns. CASA parameters are calculated based on the observed x and y coordinates of each sperm's nuclear centroid position in each video frame. In this work, we will focus on the following kinematic parameters.

- Curvilinear Velocity (VCL): Measures the total distance traveled along the sperm's actual curvilinear trajectory, expressed in microns per second.
- Straight-Line Velocity (VSL): Calculates the shortest distance between the starting and ending points of the trajectory, divided by the total time, also expressed in microns per second.
- Average Path Velocity (VAP): Represents the average velocity along a smoothed path of the sperm's trajectory, expressed in microns per second.

The tracking algorithms generally output distributions of these kinematic parameters, so variation in these parameters provide insights into the quality of the tracking, which the three experiments in the Results and Discussion showcase. When visualizing the distributions, we use 20 equally spaced bins between 0 and the maximum velocity value observed among all algorithms. When comparing distribution from different algorithms, we use the Earth Mover's Distance (EMD) between the predicted kinematic parameters and the ground truth distributions from the hand labeled data. This is implemented using the Wasserstein distance function in the SciPy Python library.

………………………..

- *Evaluating Sperm Detection and Tracking (L235)*
- *Evaluation on the Dataset (L337-44)*

We have included a new subsection in the methods with the heading **Analysis of Tracking Algorithms - Direct Metrics**. This contains information on the common multi-object tracking metrics and additional methodology components from the two sections. For convenience, it is copied in full below.

………………………..

**Analysis of Tracking Algorithms - Direct Metrics**

Because of the indirect nature of the kinematic distribution comparison, this work proposes a set of metrics that allow for direct quantitative evaluation of the tracking quality.

A microscopy video is made up of individual image frames. The task of effectively tracking sperm throughout the entirety of the video can be separated into two distinct subtasks. First, the sperm must be detected in each individual frame. Second, individual sperm must be correctly identified and linked to corresponding sperm objects in subsequent frames. This process has many similarities to the general cell tracking problem in video-microscopy of adherent somatic cells. Thus, many of the metrics used to evaluate the effectiveness of cell tracking may be adapted for sperm tracking.

Of particular interest are metrics from two common benchmarks: The Multi-object Tracking Benchmark and the Cell Tracking Challenge. General multi-object tracking has context outside of the biological realm and well-established metrics have been used such as Multi Object Tracking Accuracy (MOTA), Identification F1 Score (IDF1), and Higher Order Tracking Accuracy (HOTA). These general benchmarks are calculated based on both the accuracy of the detections (true positives, false positives, and false negatives) and the number of correctly and incorrectly linked objects between frames. For cell tracking, metrics are often defined based on the spatio-temporal graph representation of the trajectories. Each metric can be thought of as the number of steps needed to reconstruct the ground truth graph given the predicted graph. Metrics for the detection (DET) and linking (LNK) focus on accurate node and edge placement respectively, with these two being combined to determine the overall tracking performance (TRA). Another metric of interest is track fractions (TF) which describes the average length of trajectory that is correctly identified by a predicted trajectory. Additional details providing the mathematical definitions of each metric are provided in Appendix S1. For this work, we implemented the tracking metrics using the py-ctcmetrics Python library. For sperm tracking, the pairwise matching of prediction labels and ground truth labels is calculated based on distance between sperm centroids as discussed in the Results and Discussion section.

………………………..

- *“Tracking Quality’s Effect on Standard Sperm Analysis Outputs could be included in the introduction or discussion”*
- *“Unique Consideration for Evaluating Sperm Detection (L269)”*
- *“Unique Considerations for Evaluating Sperm Tracking (L301)”*
- *“The three experiments are not described in the materials.”*

Other recommendations were made about the structure and organization. We earnestly explored modifications to the structure on these points. However, we found that we could not keep a cohesive flow to the paper with these modifications. The difficulty arises with the fact that the three experiments are designed to explain and justify the need for the final presented framework, so separating certain methods and discussion pieces in isolation makes their purpose unclear. We have made modifications where possible and have also changed the Results section to **Results and Discussion** to better convey upfront the layout of our manuscript. The previous versions Discussion section is now labeled **Implications for Future Sperm Analysis**.

# Reviewer 2 Feedback

Reviewer 2’s original comments are provided in italics for convenience:

- *The manuscript presents a computational framework for evaluating sperm tracking algorithms in crowded microscopic fields. The topic is relevant and the hypothesis is sound. The manuscript is well structured and written clearly. I enjoyed reading it, up the number of videos that’s being assessed. However, there are some issues need to be clarified.*

Reviewer 2 then brought forward some specific concerns. We now address each specific concern.

- *The dataset used for validation is too small…*

The validation set remains small due to the time-intensive task of correcting tracks to obtain ground truth data. We have added language to the methodology section to note this limitation. Additionally, we have taken the time to label two additional videos to increase the overall dataset size. This brings the total sperm up to 340 and motile sperm to 264.

- *You could give the number of spermatozoa that are being evaluated instead of number of videos. This details should be given in the abstract.*

We have aggregated the data from our five videos for the experiments. We have redone Table 1, Figure 8, and Table 4 based on this recommendation. For convenience, the new tables and figures are included below.

………………………..

………………………..

Additionally, we have added counts to the VISEM data tables for more informed comparison. The count and more specific results are now indicated in the abstract. This addition is at the end of the abstract and provided below (changes highlighted in blue).:

………………………..

…In this work, we identify a framework for accurately assessing the quality of sperm trajectory tracking that is independent of standard motility measures. We utilize cell tracking metrics adapted from the more common task of tracking adherent somatic cells and propose modifications based on the unique challenges of sperm video-microscopy. We also provide a small dataset of microscopy videos that includes 340 labeled sperm trajectories to allow for future comparisons and developments. Finally, we demonstrate that variations in configuration can lead to as much as a 30% improvement on metrics, showcasing their effectiveness at analyzing tracking quality.

………………………..

- *The proposed metrics are adapted from existing tracking systems, so their novelty must be clearly defined in the methods.*

The methods now include the description of the existing metrics in a subsection called **Analysis of Tracking Algorithms - Direct Metrics** (as shown under Reviewer 1’s Feedback). Additionally, we have added the following text to the Unique Consideration for Sperm Detection to clarify the novelty and need of the modification to the association step we make (changes highlighted in blue).

………………………..

…Localization between the actual and predicted point can be quickly computed using the Hungarian algorithm with a distance cutoff. This association step based purely on centroid location is vital for effective evaluation since all standard tracking metrics require a consistent pairing mechanism between prediction labels and ground truth labels. For this work, the standard multi-object metrics (MOTA, IDF1, HOTA) and cell tracking metrics (DET, LNK, TRA, TF) are modified to include this distance-based association step. Additional details are described in the Methods section and Appendix S1.

………………………..

- *The biological implications of improved tracking must be discussed more cautiously, as they didn’t induce capacitation nor fertility.*

We have added clarifying language in section “Implications for Future Sperm Analysis”.

…Several limitations of this study should be acknowledged. First, our labeled dataset is relatively small and is simply intended as a proof-of-principle to highlight the importance of long timescale tracking. Additionally, the samples do not represent complex changes that sperm undergo during in vitro capacitation, nor was fertility of the samples directly assessed.

We again thank the reviewers for their time and suggestions that have meaningfully improved the manuscript. We have uploaded our marked-up manuscript to the submission system. As a note, we have highlighted new additions in blue, removed text in red, and sections that have been moved from one location to another in orange.
